# An efficient decision support system for leukemia identification utilizing nature-inspired deep feature optimization

**DOI:** 10.3389/fonc.2024.1328200

**Published:** 2024-03-05

**Authors:** Muhammad Awais, Md. Nazmul Abdal, Tallha Akram, Areej Alasiry, Mehrez Marzougui, Anum Masood

**Affiliations:** ^1^ Department of Electrical and Computer Engineering, COMSATS University Islamabad, Wah, Pakistan; ^2^ Department of Computer Engineering, TED University, Ankara, Türkiye; ^3^ Department of Computer Science and Engineering, University of Liberal Arts Bangladesh, Dhaka, Bangladesh; ^4^ College of Computer Science, King Khalid University, Abha, Saudi Arabia; ^5^ Department of Physics, Norwegian University of Science and Technology, Trondheim, Norway

**Keywords:** bio-inspired, CNN, transfer learning, leukemia classification, deep learning, metaheuristics optimization

## Abstract

In the field of medicine, decision support systems play a crucial role by harnessing cutting-edge technology and data analysis to assist doctors in disease diagnosis and treatment. Leukemia is a malignancy that emerges from the uncontrolled growth of immature white blood cells within the human body. An accurate and prompt diagnosis of leukemia is desired due to its swift progression to distant parts of the body. Acute lymphoblastic leukemia (ALL) is an aggressive type of leukemia that affects both children and adults. Computer vision-based identification of leukemia is challenging due to structural irregularities and morphological similarities of blood entities. Deep neural networks have shown promise in extracting valuable information from image datasets, but they have high computational costs due to their extensive feature sets. This work presents an efficient pipeline for binary and subtype classification of acute lymphoblastic leukemia. The proposed method first unveils a novel neighborhood pixel transformation method using differential evolution to improve the clarity and discriminability of blood cell images for better analysis. Next, a hybrid feature extraction approach is presented leveraging transfer learning from selected deep neural network models, InceptionV3 and DenseNet201, to extract comprehensive feature sets. To optimize feature selection, a customized binary Grey Wolf Algorithm is utilized, achieving an impressive 80% reduction in feature size while preserving key discriminative information. These optimized features subsequently empower multiple classifiers, potentially capturing diverse perspectives and amplifying classification accuracy. The proposed pipeline is validated on publicly available standard datasets of ALL images. For binary classification, the best average accuracy of 98.1% is achieved with 98.1% sensitivity and 98% precision. For ALL subtype classifications, the best accuracy of 98.14% was attained with 78.5% sensitivity and 98% precision. The proposed feature selection method shows a better convergence behavior as compared to classical population-based meta-heuristics. The suggested solution also demonstrates comparable or better performance in comparison to several existing techniques.

## 1 Introduction

Blood is a vital fluid for the human body. It performs a number of crucial physiological functions, including the distribution of oxygen and nutrients from organs to cells, delivery of waste products from cells to organs for elimination, the maintenance of the human immune system, clotting and wound healing process, and the regulation of body temperature and fluid balance. The body’s main source of blood production is the bone marrow, a spongy tissue-like structure located within the bone cavities. A complex process known as hematopoiesis involves the maturation of stem cells into other blood cell types.

**Figure 1:**
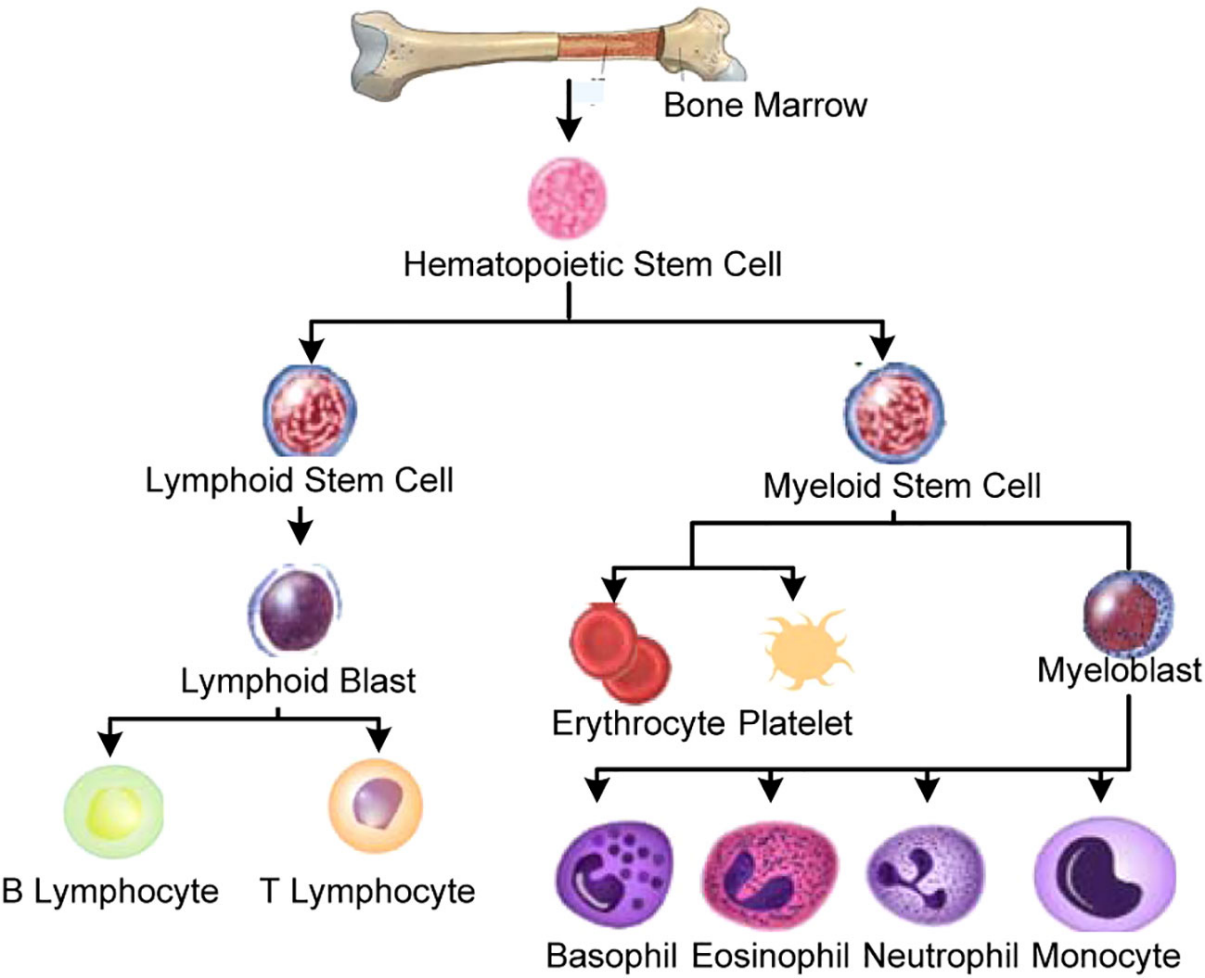
Human hematopoiesis.

**Figure 1** demonstrates the categorization of stem cells during hematopoiesis of a normal human being. The hematopoietic stem cells develop into either of two types of cells, i.e., a) lymphoid stem cells and b) myeloid stem cells. The lymphoid stem cells are then converted into the lymphoid blast, which then matures into B and T subtypes of lymphocytes. In contrast, the myeloid type of stem cells matures to synthesize erythrocytes, platelets, and various types of granulocytes (i.e., basophils, eosinophils, neutrophils, and monocytes).

**Figure 2:**
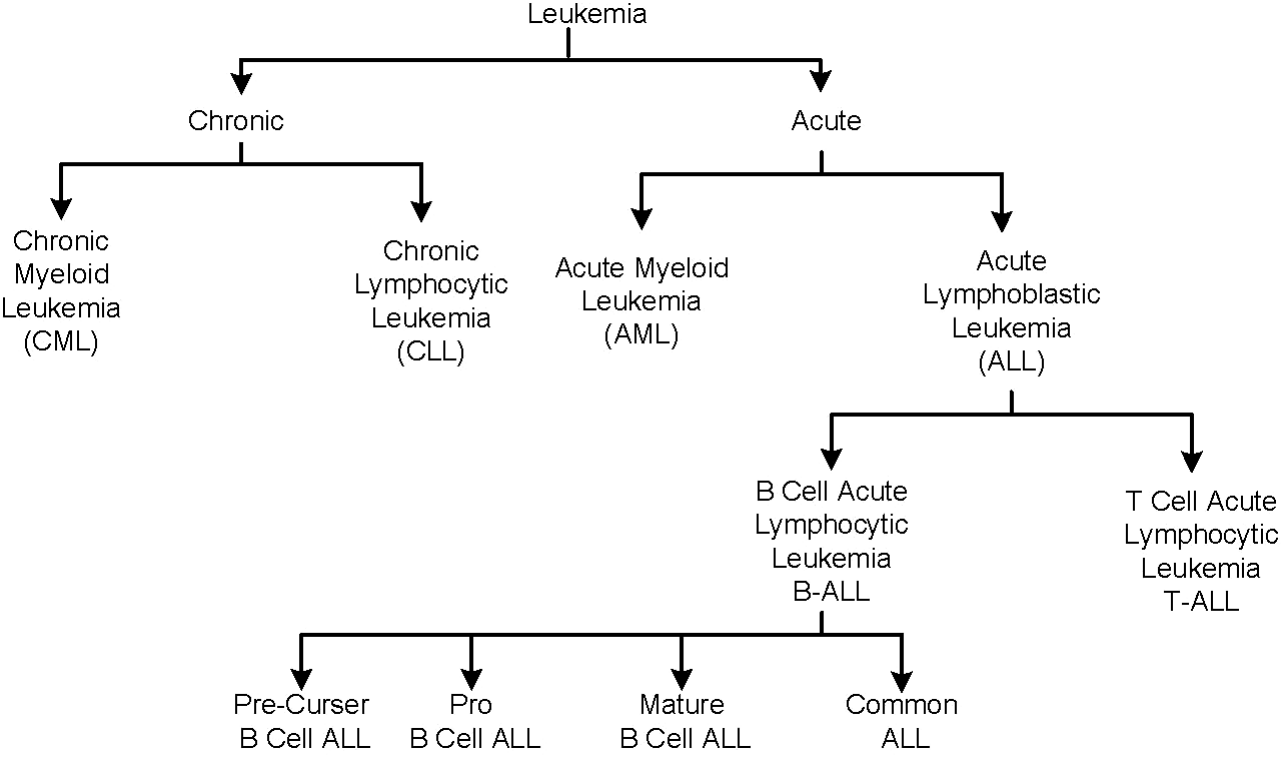
Classification of leukemia disease.

The body produces the blood cells in a controlled manner as per its requirements. Each cell type has a specific function in preserving a person’s general state of health. An abnormality in the production and structure of blood cells leads to certain medical conditions. For example, white blood cells (WBCs), also referred to as leukocytes, constitute an integral part of the general immune and inflammatory response system (1, 2). Leukemia is a blood malignancy that is caused by the unregulated production of immature leukocytes in the bone marrow. **Figure 2** shows a broad classification of leukemia, which is primarily of two types, i.e., acute and chronic, depending upon its progress rate. Chronic leukemia is slow-growing and takes months or years to manifest its symptoms, whereas acute leukemia develops rather swiftly. Each type of leukemia is further categorized on the basis of affected leukocytes. In the chronic leukemia category, chronic myeloid leukemia (CML) affects the myeloid type of cells, whereas chronic lymphocytic leukemia affects the lymphoid cells. Similarly, the acute leukemia category is further classified as acute lymphoblastic leukemia (ALL) and acute myeloid leukemia (AML) categories. ALL is further categorized into T-cell or B-cell subtypes. B-cell ALL is the most prevalent type of leukemia, impacting the B-cell lymphocytes; it is further divided into pre-curser, pro, mature, and common B-cell ALL subtypes.

The existing medical approach for leukemia diagnosis involves a series of tests, ranging from simple blood count tests to more invasive tests such as needle biopsy or bone marrow aspiration. A blood test that shows a high value of white blood cell count suggests leukemia diagnosis. An important diagnostic tool in the evaluation of leukemia is the peripheral blood smear test. It involves the smearing of blood on the slide and its visual inspection under the microscope. A blood smear of a leukemia patient shows a significant number of purple-stained lymphoblasts in the bloodstream, with poorly defined boundaries. Traditionally, hematologists perform this ocular inspection of blood smears. This manual method not only consumes much time and effort of medical experts but can also be error-prone due to several external factors. Blood analysis is usually the first step of leukemia diagnosis and is carried out in conjunction with more detailed analysis methods such as RNA sequencing and molecular genetics. Computer-aided automation of blood analysis can be a significant step in reducing the time and cost of leukemia diagnosis.

Thanks to the landmark advancement in the domain of digital electronics and imaging technologies, automated blood analysis has been made possible. In particular, computer vision-based blood disease diagnosis has seen an increased research focus in recent years. However, due to morphological similarities across various blood entities as well as their structural anomalies, accurate machine learning-based blood analysis, particularly leukemia subtype detection, is still a challenge. A breakthrough in modern computer vision approaches, i.e., deep convolutional neural networks (CNNs), has shown a promising solution for a variety of classification scenarios (3, 4). They are capable of extracting a diverse range of features from the images. However, a large and well-labeled dataset is typically required to achieve a certain acceptable accuracy level. In the biomedical domain, a detailed dataset for CNN training from scratch is not readily available. Transfer learning is an often adopted approach in which deep CNN, pre-trained on another dataset, is retrained for a specific task (5, 6). Some well-known pre-trained CNNs include ResNet (7), MobileNet (8), DarkNet (9), Inception (10), and Xception (11). Modern methods also propose ensembles of feature vectors extracted from multiple CNNs (12). Apart from the wide use of deep CNNs in the computer vision domain, one limiting factor is the very high size of their extracted feature vectors. The present focus of research is to investigate approaches to improve the accuracy of classification systems while reducing their computational complexity and memory requirements.

## 2 Literature review

Existing research on leukemia detection can be broadly categorized into two types of approaches. In the first category, the studies are included, which perform white blood cell classification as an important preliminary step. The second category of work is focused on considering the stained images containing leukemia blasts and proposing an efficient method for leukemia subtype classification. Some considerable studies from the first category are summarized as follows. Sanei and Lee proposed a method that computes eigenvectors of blood cell images using the minimization of similarity approach (13). Using the density and color information of eigencells, a Bayesian classifier was used to perform cell classification. Kumar et al. (14) used various image pre-processing techniques with a random forest classifier for blood cancer detection. Su et al. (15) suggested a segmentation method based on detecting a discriminating zone of WBCs on the hue, saturation, and intensity (HSI) space. WBC classification was performed using geometrical, color, and local directional pattern (LDP) features. Sharma et al. (16) used DenseNet121 CNN for WBC classification. Almezhghwi and Serte (17) proposed an image augmentation approach using generative adversarial networks, and classification was performed using DenseNet. Yildirim and Çinar (18) proposed Gaussian and median filtering approaches for image pre-processing. Then, multiple CNN architectures were trained for WBC classification.

**Table 1** summarizes some recently published leukemia classification methods using blood smear images containing cell blasts. Bhattacharjee and Saini (19) applied different morphological operations to perform image segmentation. This was followed by classification using multiple baseline classifiers to diagnose the presence of ALL. The proposed solution achieved the best accuracy of 95.23% with the artificial neural network (ANN) classifier. Goutam and Sailaja (20) applied K-means clustering for segmentation, followed by the local directional path technique in order to extract features, and, finally, classification using support vector machines (SVMs). The F-measure achieved by this approach was 93.44. Patel and Mishra (21) applied histogram equalization along with the zack algorithm group wbcS in the smear images. Next, various morphological features including area, color, mean, and standard deviation were extracted and classified using SVM. The overall accuracy achieved by the system was 93.57%. Rawat et al. (22) proposed a method in which leukocytes and lymphocytes were sequentially separated from other blood cells. The shape and grey-level occurrence matrices were classified using a binary SVM classifier. Mishra et al. (23) performed nucleus feature extraction from RGB images using discrete cosine transform (DCT), followed by SVM classification. Di Ruberto et al. (24) utilized a multiscale blob detection scheme followed by the watershed algorithm for segmentation and, finally, classification using CNN and SVM classifiers. The proposed solution achieves a binary classification accuracy of 94.18%. Anwar and Alam (26) proposed a three-phase filtering algorithm to perform image segmentation. Next, 16 robust features were extracted, and classification was performed using ANN and SVM classifiers, yielding a specificity of 95.41%. Bodzas et al. (25) utilized different data augmentation techniques and performed training on their custom-proposed CNN architecture to obtain an overall accuracy of 99.5% for the binary classification of leukemic cells. Batool and Byun (27) proposed a lightweight deep learning-based EfficientNet-B3 model, which employs depth-wise separable convolutions for ALL classification. The proposed method achieves an accuracy of 96.81% for leukemia subtype classification using public datasets. Elhassan et al. (28) proposed an approach of AML detection from WBC images. First, a CMYK moment-based region of interest (ROI) localization method was used, followed by deep learning-based feature extraction and classification using several baseline classifiers. The proposed system achieves the best accuracy of 97.57%. In our previous work (29), we utilized a quantum-inspired deep feature selection method for WBC classification for leukemia detection.

**Table 1:** Summary of some published studies on leukemia identification.

Author	Method	Leukemia type	Results
Bhattacharjee and Saini (19)	Morphological segmentation	ALL	Accuracy: 96.67%
	Classification: SVM		Accuracy: 90.47%
	ANN		Accuracy: 95.23%
	K-means		Accuracy: 85.71%
Goutam and Sailaja (20)	K-means clustering classification: SVM	AML	F-measure 93.44
Patel and Mishra (21)	Zack algorithm segmentation classification: SVM	ALL	Accuracy: 93.57
Rawat et al. (22)	K-means clustering classification: SVM	ALL	Accuracy: 89.8%
Mishra et al. (23)	DCT feature extraction	ALL	Accuracy: 81.66
	Classification: SVM		
Di Ruberto et al. (24)	Watershed segmentation classification: CNN, SVM	ALL	Accuracy: 94.1.8%
Bodzas et al. (25)	Classification: ANN, SVM	ALL	Specificity: 95.31%
Anwar and Alam (26)	Automated feature extraction classification: CNN	ALL	Accuracy: 99.5%

ALL, acute lymphoblastic leukemia; AML, acute myeloid leukemia; SVM, support vector machine; ANN, artificial neural network; DCT, discrete cosine transform; CNN, convolutional neural network.

Modern transfer learning-based deep CNN techniques are characterized by their ability to extract a high number of characteristics from the input images. Due to the unreasonably huge feature sets that must be stored and processed, this has enormous computational costs and memory needs (30, 31). Most frequently, a large portion of these extracted deep characteristics are redundant and provide nothing to help with categorization. By selecting just potent, discriminating characteristics, feature selection is essential to reduce the complexity of feature vectors. This shortens the processing time while simultaneously improving the accuracy of the classification system. Several studies have investigated efficient feature selection methods, which include two kinds of approaches, namely, the filter approach and the wrapper approach. The filter approach quickly converges to the critical features, but it ignores the relationship between the classification algorithm and the feature subset. The wrapper approach, in contrast, considers a tight relationship between a subset of selected features and accuracy. While nature-inspired metaheuristics have been extensively applied in a wide range of combinatorial optimization problems (32–34), they have been recently investigated for feature selection optimization (35–38).

## 3 Contributions

This work proposes an improved pipeline for ALL subtype identification. The following are the main contributions of this study.

1. First, an efficient neighborhood pixel-based contrast enhancement technique was proposed based on a differential evolution algorithm, whose parameters were optimized using a greedy metaheuristic.
2. Next, two CNNs, namely, InceptionV3 and DenseNet201, were used for feature extraction using deep transfer learning.
3. A combined feature vector was created by performing a fusion of extracted feature vectors.
4. As a main contribution, the deep feature selection problem was modeled as an optimization problem and solved using a nature-inspired Grey Wolf Optimization (GWO) algorithm. The suggested approach selects only the most pertinent features, efficiently excluding correlated and noisy information.
5. The classification performance of various baseline classifiers was validated on the selected feature set to obtain the best-performing classifiers.
6. The proposed system achieves better performance metrics as compared to several existing feature selection methods, with a significant reduction in feature vector size.

## 4 Materials and method

The key components of the suggested methodology are elaborated upon in the subsequent sections.

### 4.1 Description of datasets

**Figure 3:**
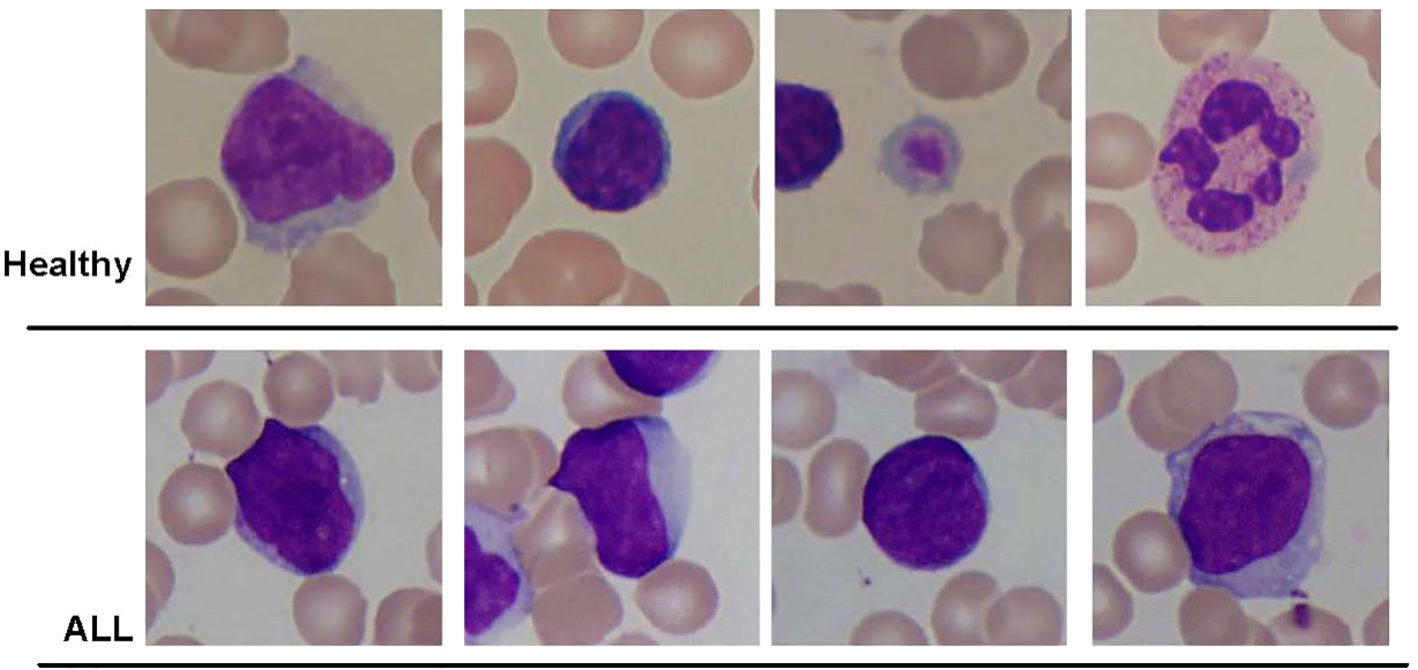
Some samples of images from ALL-IDB2 dataset of Scotti et al. (39) used in this study.

This study utilized different datasets of blood smear images. The initial dataset utilized in this study was the ALL-IDB2 dataset introduced by Scotti et al. (39). This dataset consisted of a total of 260 pictures, encompassing both healthy individuals and subjects diagnosed with ALL. The dataset was generated by employing an optical microscope that was attached to a Canon Power Shot G5 camera. The IDB2 dataset comprises images in which the region of interest has been cropped to include the area of interest for both normal and blast cells. All images were stored in the Tagged Image File Format (TIFF) and had a resolution of 2,592 pixels in width and 1,944 pixels in height. **Figure 3** displays a selection of sample images from the ALL-IDB2 dataset.

This study used multiple datasets of blood smear images. First, the ALL-IDB2 dataset was used, which was composed of 260 images of healthy and ALL subjects. The dataset was prepared using an optical microscope connected to a Canon Power Shot G5 camera. The IDB2 dataset contained images in which the area of interest of normal and blast cells was cropped as the region of interest. All images were in TIFF format with a resolution of 2,592 × 1,944 pixels. The figure demonstrates some sample images of the ALL-IDB2 dataset.

**Figure 4:**
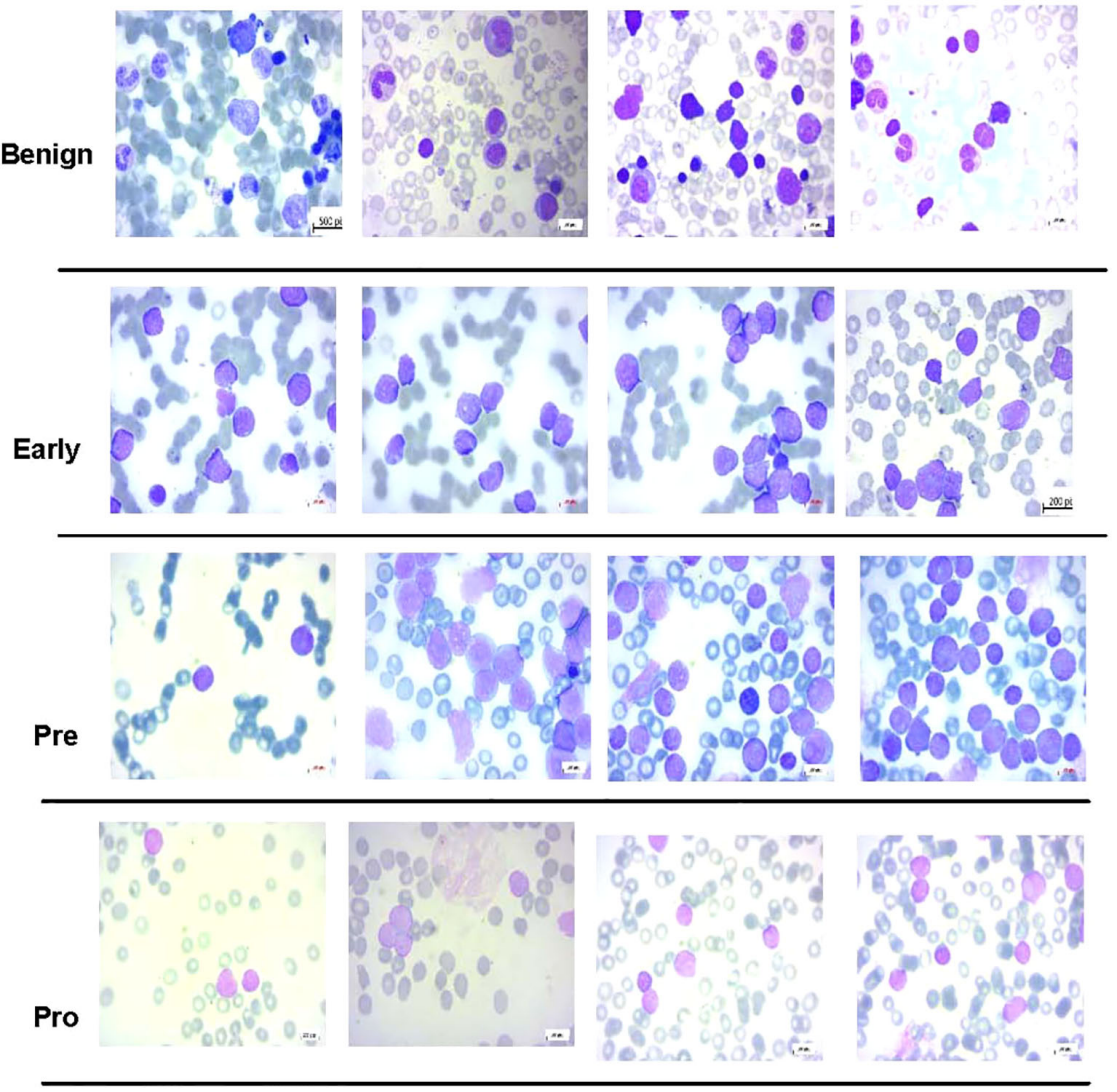
Some samples of images from acute lymphoblastic leukemia (ALL) subtype dataset (40) used in this study.

Another dataset prepared by the bone marrow laboratory of Taleqani Hospital, Iran (40) was also used. The dataset consisted of 3,242 peripheral blood smear images belonging to two classes, i.e., benign and malignant. The latter class was further divided into three sub-classes of ALL, i.e., early, precursor B-cell ALL, and pro-B-cell ALL. The images were captured using a Zeiss camera integrated with a microscope setting with ×100 magnification. The resolution of images was 224 × 224. **Figure 4** shows some sample images of the dataset of (40), whereas **Table 2** shows the class distribution.

**Table 2:** Class distribution of image dataset of Ghaderzadeh et al. (40).

Class	No. of images
Benign	512
Precursor B-cell ALL	955
Pro-B-cell	796
Early pre-B	979

ALL, acute lymphoblastic leukemia.

### 4.2 Proposed system pipeline

**Figure 5:**
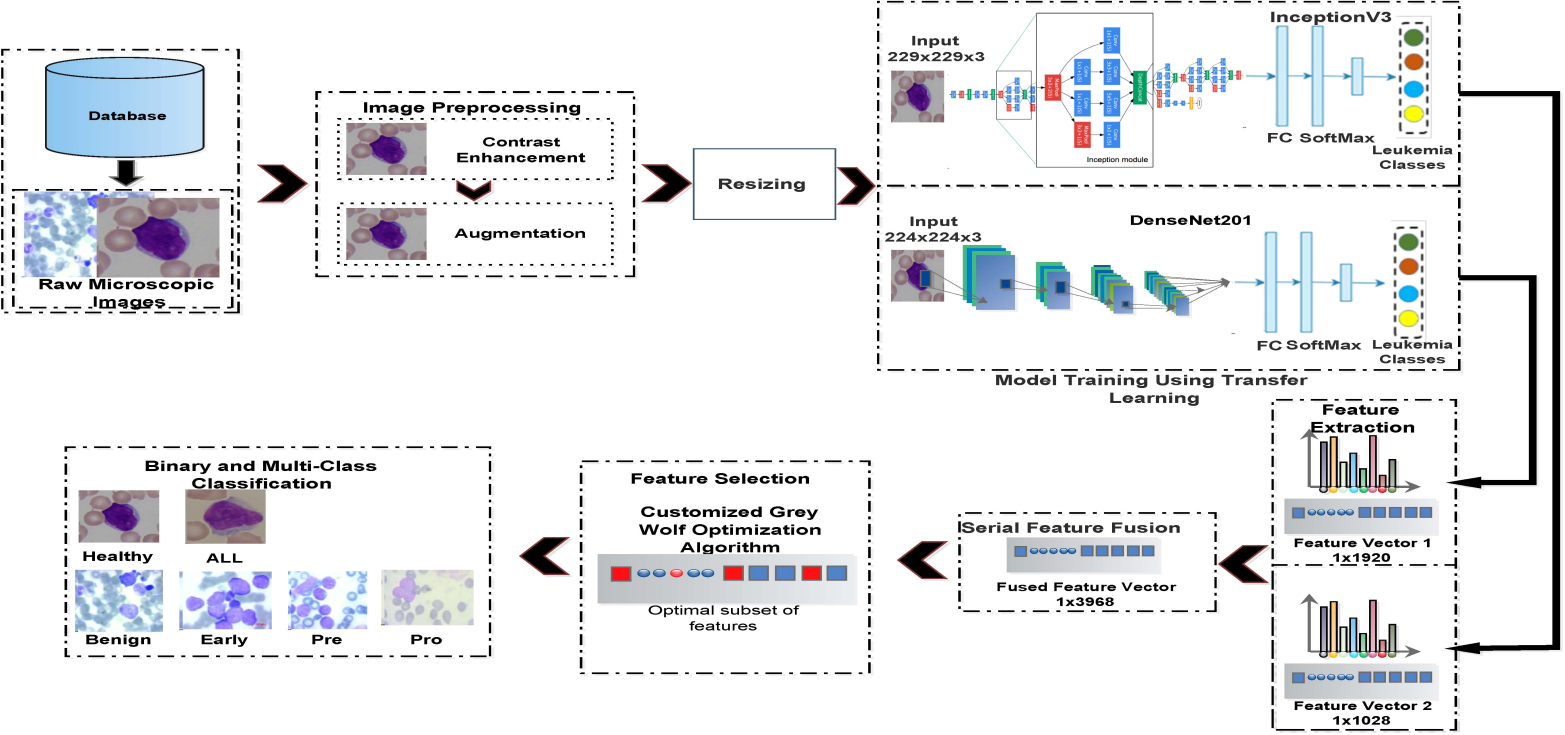
Proposed pipeline for binary and multiclass identification of acute lymphoblastic leukemia.

In **Figure 5**, a pipeline is presented for the proposed system. The main steps of computation are discussed in the following.

#### 4.2.1 Contrast enhancement

In most of the existing works, image enhancement is mainly accomplished using transforms, points, and spatial operations (41). Among the transforms, various kinds of filtering operations are included such as homomorphic or linear operations. Point operations include contrast enhancement, thresholding, and histogram adjustment. The main limitation of most of these methods is that they perform a global operation on the input image without considering region-specific contrast stretching. Spatial transformation includes neighborhood-based methods such as filtering or masking. These techniques sometimes produce unnecessary noise enhancement of images or increase the smoothness of image regions where sharpness is required (42).

In this work, we performed image contrast stretching using a greedy differential evolution approach, which consisted of the following steps.

1. Convert the input image from RGB image space to HSI image.
2. Perform contrast stretching of the I-channel of the image using the proposed greedy differential approach
3. Convert the HSI image to RGB image space.

The proposed contrast stretching method was based on neighborhood pixel transformations instead of image-wise global operators. Considering an input intensity image *I* with dimensions *M* × *N*, we used the following function for pixel-wise update based on local neighborhood (41). Mathematically, it is formulated by **Equation 1**.


(1) $$\begin{array}{l} f\left(m,n\right)=\left(\frac{\mu .\delta }{\beta +\sigma_{p}\left(m,n\right)}\right)\left[I\left(m,n\right)-\gamma \mu_{p}\left(m,n\right)\right]\\ +\mu_{p}{\left(m,n\right)}^{\alpha }\;\forall m\in M,n\in N, \end{array}$$

where *I*(*m*, *n*) is the current pixel value of the intensity image with coordinates *m* and *n*, *µ* is the global mean of the image, *µ_p_* (*m*, *n*) is the local mean, and *σ_p_* (*m*, *n*) is the local variance of a window comprising of *p* × *p* neighborhood pixels around the central pixel *I*(*i*, *j*). Munteanu and Rosa (41) used the non-zero constants *α*, *β*, and *γ*. The non-zero value of *β* allows to have zero standard deviation *σ_p_* (*i*, *j*) of the pixel window. The final term of Equation 2 was added to achieve smoothness while preserving the brightness.

In this work, we performed automatic estimation of decision variables *α*, *β*, *γ*, and *δ* using a meta-heuristic algorithm. The following objective function was used in **Equation 2**:


(2) $$F\left(I^{*}\right)=\frac{log(log(G\left(I^{*}\right))\times n_{e}\left(I^{*}\right)\times H\left(I^{*}\right)}{M\times N},$$

where *I* ^∗^ denotes an enhanced intensity image obtained using (2) on the input intensity image, and *n_e_* (*I* ^∗^) and *H*(*I* ^∗^), respectively, denote the number of pixels on the boundary and value of entropy of *I* ^∗^. The value of function *G*(*I* ^∗^) was obtained by applying a Sobel kernel on the enhanced intensity image *I* ^∗^.

#### 4.2.2 Optimization of decision variables using differential evolution

The estimation of variables *α*, *β*, *γ*, and *δ* was performed using bounded exploration of search space. First, a population matrix *P* consisting of *N_p_* row vectors was generated, where each vector was composed of four variables, i.e., *α*, *β*, *γ*, and *δ*. Each entity of the population matrix was generated randomly, as **Equations 3**–**6** (43).


(3) $$\begin{array}{l} P\left(i,j\right)=l_{b}+r_{1}\times \left(u_{b}-l_{b}\right),\\ \forall i\in \left\{1,\ldots ,N_{p}\right\},j\in \left\{1,\ldots ,4\right\}, \end{array}$$

where *l_b_* and *u_b_* are respectively the minimum and maximum values of the decision variable as given in **Table 3**, and *r*_1_ denotes a random number in [0, 1]. All vectors of population *P* then participated in computing the value of intensity transformation function *f*(*i*, *j*) of Equation 2 and objective function *F*(*I* ^∗^) of Equation 2. The vector yielding the maximum value of the objective function was selected as the population best, i.e., *P_b_*.

**Table 3:** Minimum and maximum values of decision variables used in differential evolution.

Decision variable	Minimum value (*l_b_*)	Maximum value (*u_b_*)
*α*	0	1.6
*β*	0	0.5
*γ*	0	0.8
*δ*	0.5	1.5

In the next step, each population vector *P_i_ i* ∈{1, …, *N_p_*} underwent mutation operation to generate its corresponding mutation vector *M_i_* such that (43)


(4) $$M_{i}\left(t\right)=P_{b}\left(t\right)+A\left(P_{r1}-P_{r2}\right)\;+A\left(P_{r3}-P_{r4}\right),$$

where *t* denotes the value of the current iteration, *P_b_* (*t*) denotes the iteration’s best individual vector, and scaling factor *A* is a random number in [0, 1]. The indices of population vectors (i.e., *r*1, *r*2, *r*3, and *r*4) were randomly chosen such that they are all distinct from each other and the index *i* of the current population vector.

The population vector *P_i_* and its corresponding mutation vector *M_i_* then underwent binomial crossover operation to generate vector *C_i_* such that (43)


(5) $$C_{i}\left(t\right)=\left\{\begin{array}{cc} P_{i}\left(t\right) & i\neq x_{1}\;\&\;r>x_{2}\\ C_{i}\left(t\right) & i=x_{1}\text{ or }r\leq x_{2} \end{array}\right\},$$

where *x*_1_ is a randomly generated index within the interval [1, *N_p_*] and *x*_2_ is a random number in [0, 1]. Next, all decision variables each vector *C_i_* (*t*) are bounded within limits *l_b_* and *u_b_*. In differential evolution, a greedy selection of survival of the fittest was carried out using the following criteria to update the population matrix (43).


(6) $$P_{i}\left(t+1\right)=\left\{\begin{array}{cc} P_{i}\left(t\right) & \text{ if }F\left(C_{i}\right)<F\left(P_{i}\right)\\ C_{i}\left(t\right) & \text{otherwise} \end{array}\right\},$$

where *F*(*P_i_*) and *F*(*C_i_*) denote the cost of the objective function (2) using the vectors *P_i_* and *C_i_*, respectively. This completes one iteration of the algorithm, which was repeated for *T* iterations.

#### 4.2.3 Feature extraction

The contrast-enhanced images of datasets were used in the feature extraction step. For this purpose, we employed transfer learning as a feature extraction using two well-known deep CNNs, namely, InceptionV3 and ResNetV2.

InceptionV3 is a deep CNN that belongs to the Inception family of CNNs. It is pre-trained on the ImageNet database (44) consisting of 1,000 object classes. The network has 316 layers and 350 connections (45). The size of the first layer, i.e., the input layer, is 299 × 299 × 3. A convolution layer consists of different filters and stride sizes. Each convolution layer also incorporates batch normalization and ReLU layers for adding non-linearity. A pooling layer is also added between the convolution layers to obtain active neurons. The addition of Inception modules is a distinguishing characteristic of this network. They are designed for multiscale feature extraction. Each inception module is built using multiple parallel convolution layers with different filter sizes and a pooling layer. The outputs of these layers are concatenated along the depth dimension.

To use InceptionV3 for feature extraction, its last learnable layer, “predictions”, was replaced with a fully connected layer, which had outputs matching the number of classes of our datasets [i.e., two classes for ALL-IDB2 and four classes for the dataset of (40)]. The softmax layer named “predictions softmax” was replaced by the new softmax layer. A label-free classification layer was added to the network, which replaced the “ClassificationLayer predictions” layer. Prior to the network training, the dataset image resizing was performed per the requirement of the network’s input layer. Then, specific augmentation steps were performed. The activations were applied on the “avg pool” layer, and a feature vector of length 2,048 was obtained per image of the training and testing datasets.

DenseNet201 (46) has a depth of 201 layers and was initially trained on the ImageNet (44) dataset. The DenseNet201 is based on the idea of layer concatenation; i.e., each layer obtains data from all of its previous layers and transfers its computed feature maps to all its next layers. As a result, a thinner and more compact network is realized, which is computationally efficient and achieves considerable memory savings.

To use DenseNet201 as a feature extractor, its “fc1000” layer was substituted with a fully connected one that contains an equal number of classes from our datasets. Similarly, a new softmax and classification layer without labels was inserted in the network in place of their respective layers. After performing image resizing and augmentation, feature activation was applied on the global average pool layer, and a feature vector of size 1,920 was extracted per image of the dataset.

#### 4.2.4 Feature fusion

In this work, we performed a simple horizontal concatenation of the individual feature vectors extracted from the abovementioned deep CNNs and formed a fused feature vector of size 1 × (*a* + *b*), where *a* = 2,048 and *b* = 1,920 are the number of features extracted from InceptionV3 and DenseNet201 networks, respectively.

#### 4.2.5 Meta-heuristic for feature selection

The combined feature vector obtained from the transfer learning steps above has a considerably large size. Directly using the extracted fused feature vector to train the baseline classifiers requires a huge amount of processing power and memory. As a main contribution, this work modeled the optimization problem of feature selection with the objective of maximizing classification accuracy with the minimum feature set. The optimization problem was then solved with the help of a population-based meta-heuristic named Grey Wolf Algorithm. This technique (47) mimics the hunting behavior of grey wolves. A pack of grey wolf apex predators consists of an average of five to 12 individuals. The grey wolf population is composed of four distinct individuals categorized as alpha, beta, delta, and omega, based on their dominant order. The alpha wolf is the individual within a population that holds the highest rank and assumes the role of decision-maker and dominant figure. The subsequent position in the dominance hierarchy is occupied by the beta wolf. It is subordinate to the alpha and helps in the decision-making. The delta wolf ranks third in the hierarchy and only dominates the least significant omega group.

In the mathematical framework of GWO, the most optimal solution is referred to as the alpha wolf (*α*). Subsequently, the second and third most optimal solutions are denoted as the beta (*β*) and delta (*δ*) wolves, respectively. The main steps of grey wolf hunting are as follows:

- Search the prey (exploration).
- Encircle the prey.
- Attack the prey (exploitation).

The prey encircle behavior of a pack of wolves is mathematically modeled as defined in **Equations 7**–**18**.


(7) $$D=\left|C.X_{p}-V\left(t\right)\right|,$$

(8) $$V\left(t+1\right)=V_{p}\left(t\right)-A.D,$$

where *V_p_* denotes the prey vector position at iteration *t*, *V* (*t*) is the current position of the grey wolf, A and C are the vectors of coefficients:


(9) $$A=2.a.x_{1}-a,$$

(10) $$C=2.x_{2}$$

where *x*_1_ and *x*_2_ are vectors containing random vectors in [0, 1], and *a* is the encircling coefficient that mimics the encircling behavior by decreasing linearly from 2 to 0, which is linearly decreased from 2 to 0 with iterations as (47)


(11) $$a=2-2\left(\frac{t}{t_{max}}\right)$$

where *t_max_* is the maximum number of algorithm iterations. During an iteration *t*, each wolf updates its position using the *α*, *β*, and *δ* wolves such that (47)


(12) $$V\left(t+1\right)=\frac{V_{1}+V_{2}+V_{3}}{3}$$

(13) $$V_{1}=\left|V_{\alpha }-A_{1}.D_{\alpha }\right|$$

(14) $$V_{2}=\left|V_{\beta }-A_{1}.D_{\beta }\right|$$

(15) $$V_{3}=\left|V_{\delta }-A_{1}.D_{\delta }\right|$$

where *V_α_*, *V_β_*, and *V_δ_* denote the position vectors of *α*, *β*, and *δ* wolves, respectively, at iteration *t*; *A*_1_, *A*_2_, and *A*_3_ are computed using Equation 9. The vectors *D*, *D_β_*, and *D_δ_* are computed as


(16) $$D_{\alpha }=\;\left|C_{1}V_{\alpha }-V\right|$$

(17) $$D_{\beta }=\;\left|C_{1}V_{\beta }-V\right|$$

(18) $$D_{\delta }=\;\left|C_{1}V_{\delta }-V\right|$$

The coefficients *C*_1_, *C*_2_, and *C*_3_ are computed using Equation 10. The original GWO algorithm of Mirjalili et al. (47) is generally applicable to continuous optimization problems with variables *X* ∈ ℛ.

##### 4.2.5.1 Binary Grey Wolf Algorithm

This work used a binary GWO algorithm of Emary et al. (48), in which the position update of the wolf is determined using the crossover operation of individual genes, and mathematically formulated by **Equations 19**–**23**. 


(19) $$V\left(t+1\right)=\left(V_{1}⊗V_{2}⊗V_{3}\right)$$

where *V*_1_, *V*_2_, and *V*_3_ are binary vectors for dimension *d_max_* and computed as


(20) $$V_{i}^{d}=\left\{\begin{array}{ll} 1, & \text{if }\left({\text{Δ}}_{j}^{d}+V_{j}^{d}\right)\geq 1\\ 0, & \text{ otherwise } \end{array}\right\}\;\;\;,\forall d\in D$$

For i=1,2,3 , $V_{j}^{d}$ is equal to $V_{\alpha },V_{\beta }$ , and $V_{\delta }$ , whereas ${\text{Δ}}_{j}^{d}$ is equal to ${\text{Δ}}_{\alpha }^{d},{\text{Δ}}_{\beta }^{d}$ , and ${\text{Δ}}_{\delta }^{d}$ respectively. ${\text{Δ}}_{\delta }^{j}$ is computed as (48):


(21) $${\text{Δ}}_{j}^{d}=\left\{\begin{array}{ll} 1, & \text{if }S_{j}^{d}\geq r_{4}\\ 0, & \text{otherwise } \end{array}\right\}$$

where *r*_4_ is the vector of random numbers in [0, 1]. The continuous step size $S_{j}^{d}$ is computed as (48)


(22) $$S_{j}^{d}=\frac{1}{exp\left(-10\left(A_{1}^{d}.D_{j}^{d}-0.5\right)\right)+1}$$

$D_{j}^{d}$ is equal to $D_{\alpha },D_{\beta }$ , and $D_{\delta }$ , respectively, for i=1, 2 , and 3. $A_{1}^{d}$ is computed using Equation 9, whereas $D_{j}^{d}$ is computed using Equations 16 – 18. The new position of the wolf is updated using the following crossover operation (48).


(23) $$V^{d}\left(t+1\right)=\left\{\begin{array}{ll} V_{1}^{d}, & \text{if }r_{6}<\frac{1}{3}\\ V_{2}^{d}, & \text{if }\frac{1}{3}\leq r_{6}<\frac{2}{3}\\ V_{3}^{d}, & \text{ otherwise} \end{array}\right\}$$

where *r*_6_ is a random variable that follows a uniform distribution in the interval [0, 1].

##### 4.2.5.2 Wrapper feature selection using binary GWO

This study presents the application of the binary GWO method for the purpose of deep feature selection within the leukemia classification pipeline. The computational steps of the suggested feature selection strategy are presented in **Algorithm 1**.

The main inputs to the binary GWO algorithm include the fused feature matrix F , the vector *L*, which contains the labels of the training image set; the maximum count of iterations *t_max_* the size of grey wolf population *n_p_*; and dimension size *d_max_*, which represents the total number of variables (features) of each wolf (solution) of population. The size of matrix F is *n_t_* × *d_max_*, where *n_t_* and *d_max_* respectively denote the number of training images and the dimension of fused feature vector per image.

Phase 1 initializes the main parameters including iteration counter *t*, and alpha, beta, and delta grey wolves *X_α_*, *X_β_*, and *X_δ_* along with their fitness values *f_α_*, *f_β_*, and *f_δ_*, respectively. In Phase 2, an initial population is generated and stored in matrix X of size *n_p_* × *d_max_*. The *randn*(1, *n_p_*, 1: *d_max_*) function generates a matrix of dimensions *n_p_* ×*d_max_* of binary values of uniform distribution in [0, 1]. The execution phase of the GWO algorithm proceeds in Steps 6–75. The while loop is executed for *t_max_* iterations. In an iteration, first, a prey is extracted from the population matrix (Step 8), and its fitness is evaluated (Step 9). The *Fitness* function receives three inputs, namely, the fused feature set F , the vector *L* of labels, and one member of the population, i.e., a binary vector *X*. In the *Fitness* function routine, Steps 85–86 obtain the features from F , which are indexed by non-zero values of *X*. The updated feature matrix F _2_ is then divided into testing and training parts. In Steps 87–93 of the *Fitness* function, the classification error of the K-nearest neighbor (KNN) classifier is used as a fitness value (cost). This value is then used to update the alpha, beta, and delta *X_α_*, *X_β_*, and *X_δ_* grey wolf vectors, respectively, in Steps 10–23 of the main function. Steps 26–74 of the main routine perform the position update of each grey wolf of the population according to Equations 19, 20 of the binary GWO algorithm. After the execution of the while loop for *t_max_* iterations, the global best solution, i.e., alpha wolf *X_α_*, contains the indices of features to be selected from the fused feature vector.

ALGORITHM 1. Feature selection based on binary GWO algorithm 
  1**: External Inputs:**
F

*, L,d_max_,t_max_,n_p_
*

2: **Phase 1: Initialization of Main Parameters** *t* ← 1,
 
  *V_α_
*(1,1: *d_max_
*) ← 0*, f_α_ *← ∞

  *V_β_
*(1,1: *d_max_
*) ← 0*, f_β_ *← ∞

  *V_δ_
*(1,1: *d_max_
*) ← 0*, f_δ_ *← ∞

3: **Phase 2: Generate Initial Population of Grey Wolves**
4: (1: *n_p_
*,1: *d_max_
*) ← *randn*(1: *n_p_
*,1: *d_max_
*)

5: **Execution**

6: **while** *i< t_max_ *
**do**

7:  **for** *j* = 1: *n_p_ *
**do**

8:   *V* ← (*j*,1: *d_max_
*)
  9:   *f* ←*Fitness* (
F

*, L, V*)
10:  **if** *f< f_α_ *
**then**

11:   *Vβ*←*Vα*

12:   *fβ* ←*fα*

13:   *V_α_ *←*V*

14:   *f_α_ *← *f*

15:  **else if** *f< f_β_ *
**then**

16:   *Vδ* ← *Vβ*

17:   *fδ* ← *fβ*

18:   *V_β_
*←*V*

19:   *f_β_ *← *f*

20:  **else**

21:   *V_δ_ *← *V*

22:   *f_δ_ *← *f*

23:  **end if**

24: **end for**

25:  **Population Update**

26: **for** *j* = 1: *n_p_ *
**do**

27:  **for** *d* = 1: *d_max_ *
**do**

28:   
$a\leftarrow 2-2\frac{i}{t_{max}}$


29:   *a*
_1_←2.*a.rand*(1, 1)-*a*

30:   *a*
_2_←2.*a.rand*(1, 1)-*a*

31:   *a*
_3_←2.*a.rand*(1, 1)-*a*

32:   *c*
_1_←2*.rand*(1, 1)-*a*

33:   *c*
_2_←2*.rand*(1, 1)-*a*

34:   *c*
_3_←2*.rand*(1, 1)-*a*

35:   
$D_{\alpha }\left(1,d\right)\leftarrow \left|c_{1}.V_{\alpha }\left(1,d\right)-V\left(j,d\right)\right|$


36:   
$D_{\beta }\left(1,d\right)\leftarrow \left|c_{1}.V_{\alpha }\left(1,d\right)-V\left(j,d\right)\right|$


37:   
$D_{\delta }\left(1,d\right)\leftarrow \left|c_{1}.V_{\alpha }\left(1,d\right)-V\left(j,d\right)\right|$


38:   
$S_{1}\left(1,d\right)\leftarrow \frac{1}{1+exp\left(-10\left(a_{1}.D_{\alpha }\left(1,d\right)-0.5\right)\right)}$


39:   
$S_{2}\left(1,d\right)\leftarrow \frac{1}{1+exp\left(-10\left(a_{2}.D_{\beta }\left(1,d\right)-0.5\right)\right)}$


40:   
$S_{3}\left(1,d\right)\leftarrow \frac{1}{1+exp\left(-10\left(a_{3}.D_{\delta }\left(1,d\right)-0.5\right)\right)}$


41:   **if** *S*
_1_(1*,d*) ≥ *randn*(1,1) **then**

42:     Δ_1_(1*,d*) ← 1
43:   **else**

      Δ_1_(1*,d*) ← 0
44:   **end if**

45:   **if** *S*
_2_(1*,d*) ≥ *randn*(1,1) **then**

46:     Δ_2_(1*,d*) ← 1
47:   **else**

      Δ_2_(1*,d*) ← 0
48:   **end if**

49:   **if** *S*
_3_(1*,d*) ≥ *randn*(1,1) then
50:     Δ_3_(1*,d*) ← 1
51:   **else**

      Δ_3_(1*,d*) ← 0
52:   **end if**

53:   **if** (*V_α_
*(1*,d*) + Δ_1_(1*,d*)) ≥ 1 **then**

54:     *Z*
_1_(1*,d*) ← 1
55:   **else**

      *Z*
_1_(1*,d*) ← 0
56:   **end if**

57:   **if** (*V_β_
*(1*,d*) + Δ_2_(1*,d*)) ≥ 1 **then**

58:     *Z*
_2_(1*,d*) ← 1
59:   **else**

      *Z*
_2_(1*,d*) ← 0
60:   **end if**

61:   **if** (*V_δ_
*(1*,d*) + Δ_3_(1*,d*)) ≥ 1 **then**

62:     *Z*
_3_(1*,d*) ← 1
63:   **else**

      *Z*
_3_(1*,d*) ← 0
64:   **end if**

65:   *r* ← *rand*(1,1)
  66:   **if** 
$r<\frac{1}{3}$
 **then**

67:     
$V\left(j,d\right)\leftarrow Z_{1}\left(1,d\right)$


68:   **else if** 
$r\geq \frac{1}{3}andr<\frac{2}{3}$
 **then**

69:     
$V\left(j,d\right)\leftarrow Z_{2}\left(1,d\right)$


70:   **else**

71:     
$V\left(j,d\right)\leftarrow Z_{3}\left(1,d\right)$


72:   **end if**

73:  **end for**

74: **end for**

75: **end while**

76: **Select Features**

77: *I* ← 1: *d_max_
*

78: *S_F_ *← *I*((*V_α_ *== 1))

  **OUTPUT:** *S_F_
*

79: **Function:** *Fitness*

80: **Inputs:** *V,L*,
F


81: **Parameters:** *k* = 5*,h_o_ *= 0.2*,α*
_1 = _0.99*,α*
_2 = _0.01

82: **if** (sum(*V* == 1) == 0) **then**

83:  Γ = 1

84: **else**

85:  
$F_{2}\leftarrow F\left(:,\left(V==1\right)\right)$


86:  
$A_{train},L_{train},A_{test},L_{test}\leftarrow partition\left(F_{2},L,h_{o}\right)$


87:  
$Model\leftarrow trainKNN\left(A_{train},L_{train},k\right)$


88:  
$L_{pred}\leftarrow predict\left(Model,A_{test}\right)$


89:  
$a\leftarrow sum\left(L_{pred}==L_{test}\right)/length\left(L_{test}\right)$


90:  
e←1−a


91:  
$q_{s}\leftarrow sum\left(a==1\right)$


92:  
$q_{t}\leftarrow length\left(V\right)$


93:  
$\Gamma \leftarrow \alpha_{1}\times e+\alpha_{2}\times \left(\frac{q_{s}}{q_{t}}\right)$


94: **end if**

95: **Return:** Γ

#### 4.2.6 Classification

The set of selected features from the binary GWO algorithm was then used along with the label L for training and classification of outer classifiers. Multiple classifiers were used in this work, and the best-performing classifiers were selected.

## 5 Results and discussion

The proposed decision support system for leukemia identification was implemented on an Intel Core i5 CPU with and 64-bit Windows 10 operating system and 16GB RAM.

### 5.1 Experiment 1: binary classification

First, the proposed pipeline was implemented for binary detection of leukemia using the ALL-IDB2 dataset. The classification performance of CNN was influenced by the quality and size of the training dataset. A small dataset leads to overfitting and poor generalization of the model. Hence, augmentation of contrast stretched ALL-IDB2 dataset was performed using the operations of random rotation, flipping, intensity modification, and brightness correction. **Table 4** shows the class distribution of ALL-IDB2 as a result of augmentation. In the next step, the augmented dataset was divided into training and test parts with a 70:30 split ratio, as shown in **Table 5**. Then, the training dataset was used for transfer learning of InceptionV3 and DenseNet201 models with parameters listed in **Table 6**.

**Table 4:** Class distribution of ALL-IDB2 dataset before and after augmentation.

Class	Frequency	
	Before	After
Healthy	130	593
ALL	130	601

ALL, acute lymphoblastic leukemia.

**Table 5:** Training and testing ALL-IDB2 dataset for binary classification of leukemia.

Class	Training Images	Testing Images	Total
Healthy	415	178	593
ALL	420	181	601
Total	835	359	1,194

ALL, acute lymphoblastic leukemia.

**Table 6:** Parameter settings for training of InceptionV3 and DenseNet201 models.

Parameter	Value	Parameter	Value
Kernel type	sdgm	Max epochs	10
Initial learning rate	1× 10^−4^	Environment	Auto
Validation frequency	30	Stride size	1
Mini-batch size	20	Dropout rate	0.1

InceptionV3 and DenseNet201 return deep feature vectors of sizes 2,048 and 1,920, respectively, which are horizontally concatenated to obtain a fused feature vector of size 3,968. This vector is then subjected to the proposed feature selection step using the GWO algorithm. After a fixed number of iterations, the GWO algorithm returns its best solution, i.e., a reduced vector of the most important selected features, which are then used to train several baseline classifiers with multiple settings of their kernel. **Table 7** shows the performance results of the proposed binary classification pipeline. The KNN classifier with cosine kernel achieves the best performance metrics with a reduced feature vector of 797 features, which is approximately 80% smaller than the original fused feature vector of size 3,986. The confusion matrix of the KNN cosine classifier is demonstrated in **Table 8**.

**Table 7:** Results of binary classification of leukemia on ALL-IDB2 dataset.

Classifier	Selected feature vector size	Accuracy %	Sensitivity	F1 score	Precision	Recall
KNN cosine KNN coarse KNN cubic KNN fine	797	98.1 97.8 97.9 97.5	0.981 0.971 0.981 0.964	0.987 0.981 0.976 0.965	0.98 0.972 0.972 0.989	0.987 0.972 0.972 0.977
SVM (regression) SVM (Gaussian) SVM (quadratic)		85.2 86.4 72.2	0.887 0.894 0.734	0.842 0.891 0.741	0.890 0.901 0.882	0.80 0.86 0.72
Decision tree (medium)		72.4	0.742	0.725	0.73	0.726
NN wide		94.8	0.925	0.911	0.932	0.951

KNN, K-nearest neighbor; SVM, support vector machine; NN, neural network.

**Table 8:** Confusion matrix of binary classification experiment of ALL-IDB2 with KNN-cosine classifier.

		Predicted class		TPR	FNR
		ALL	Healthy		
True Class	ALL	177	4	97.7%	2.2%
	Healthy	2	176	98.8%	1.2%

TPR, true-positive rate; FNR, false-negative rate; KNN, K-nearest neighbor; ALL, acute lymphoblastic leukemia.

**Figure 6:**
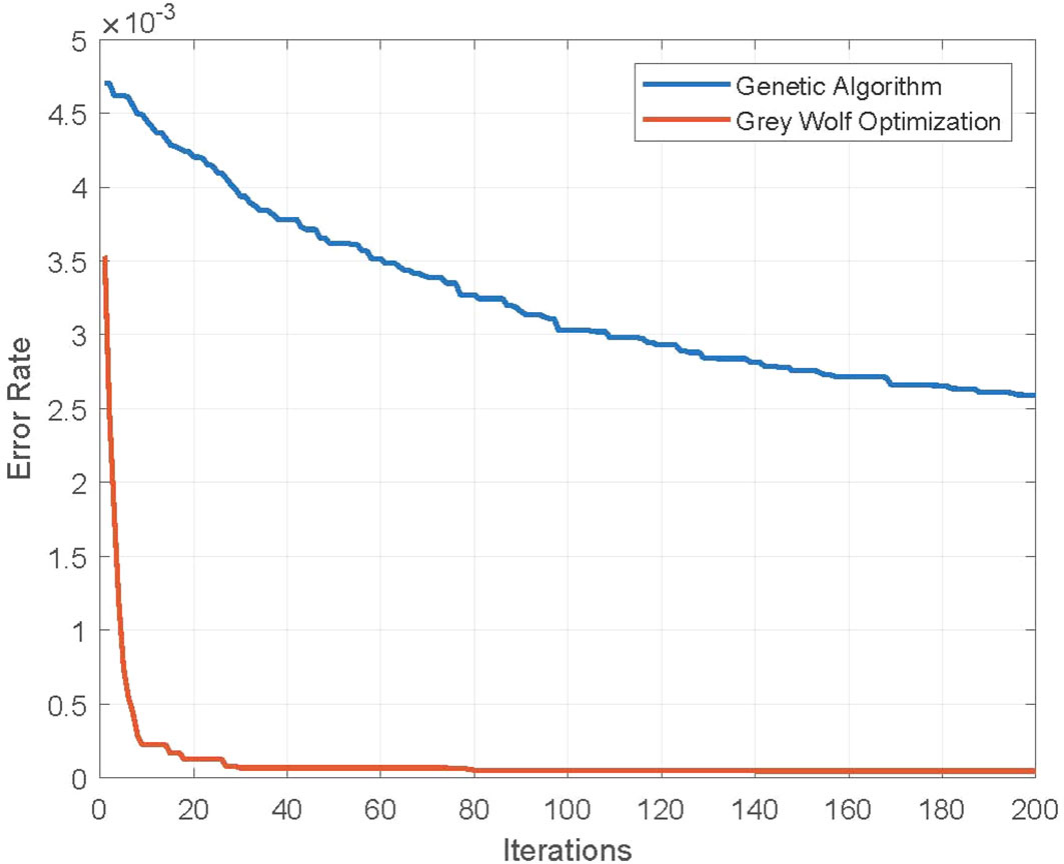
Error rate of feature selection using genetic and Grey Wolf Optimization algorithms. Population size *n_p_* = 20.

In **Figure 6**, the error rate of the GWO algorithm is plotted along with the standard genetic algorithm (GA), as a function of iterations with a constant value of population size *n_p_* = 20. A better convergence behavior is demonstrated by the GWO algorithm as compared to GA, which reveals that GWO performs better exploration of feature search space.

### 5.2 Experiment 2: leukemia subtype classification

In the second step, the proposed pipeline was implemented for multiclass problems, i.e., leukemia subtype identification using the dataset of Ghaderzadeh et al. (40). As discussed earlier, the dataset consists of four classes, i.e., benign, precursor, pro-B-cell, and early pre-B. Following the contrast stretching phase (Section 4.2.1) on the dataset, the augmentation was carried out using the same methodology as binary classification. The class distribution of the augmented dataset is shown in **Table 9**. Next, with a splitting ratio of 70:30, the training and testing parts of the dataset were extracted as shown in **Table 10**.

**Table 9:** Class distribution of dataset of Ghaderzadeh et al. (40) before and after augmentation.

Class	Frequency	
	Before	After
Benign	512	1,024
Precursor	955	1,000
Pro-B-cell	796	1,050
Early pre-B	979	1,020

**Table 10:** Class distribution of training and testing parts of dataset of Ghaderzadeh et al. (40) for leukemia subtype classification.

Class	Training images	Testing images	Total
Benign	716	308	1,024
Precursor	700	300	1,000
Pro-B-cell	735	315	1,050
Early pre-B	714	306	1,020
Total	2,149	1,229	4,094

In **Table 11**, the leukemia subtype classification results are presented. Several classifiers with various kernels were tested. The performance metrics, i.e., accuracy, precision, recall, sensitivity, specificity, and F1 score, were computed through macro averaging of the individual class metrics. In this case, the SVM classifier with Gaussian kernel achieved the best average accuracy of 98.05%, whereas the maximum average accuracy values achieved by KNN, decision tree, and neural network (NN) classifiers were 97.9%, 82.4%, and 95.8%, respectively. The testing confusion matrix with the SVM Gaussian classifier is demonstrated in **Table 12**, whereas the class-wise statistics are mentioned in **Table 13**. The maximum accuracy of 98.66% was achieved by the pro-B-cell class, whereas all other classes achieved an accuracy of above 90%.

**Table 11:** Results of leukemia subtype classification using the dataset of Ghaderzadeh et al. (40).

Classifier	Selected feature vector size	Accuracy %	Sensitivity	F1 score	Precision	Recall
KNN cosine KNN coarse KNN cubic KNN fine	797	97.9 97.6 97.4 96.4	0.783 0.781 0.709 0.771	0.78 0.78 0.71 0.771	0.978 0.976 0.974 0.96	0.783 0.781 0.974 0.771
SVM (regression) SVM (Gaussian) SVM (quadratic)		85.2 98.14 96.2	0.721 0.785 0.638	0.642 0.78 0.61	0.890 0.981 0.96	0.659 0.785 0.61
Decision tree (medium)		82.4	0.68	0.689	0.82	0.826
NN wide		95.8	0.71	0.86	0.952	0.086

KNN, K-nearest neighbor; SVM, support vector machine; NN, neural network.

**Table 12:** Confusion matrix of leukemia subtype identification using SVM Gaussian classifier on dataset of Ghaderzadeh et al. (40).

			Predicted class			TPR	FNR
		Benign	Precursor	Pro-B-cell	Early pre		
True Class	Benign	1,004	11	5	4	98.04%	1.9%
	Precursor	5	982	11	2	98.2%	1.8%
	Pro-B-cell	2	2	1,036	10	98.6%	1.4%
	Early pre	6	13	5	996	97.67%	2.35%

TPR, true-positive rate; FNR, false-negative rate; SVM, support vector machine.

**Table 13:** Statistics of individual classes using SVM Gaussian kernel.

Class	Accuracy %	Sensitivity %	Precision %	Recall
Benign	98.04	98.72	98.72	0.9804
Precursor	98.2	97.42	97.42	0.982
Pro-B-cell	98.66	98.01	98.01	0.986
Early pre	97.64	98.41	98.41	0.976

SVM, support vector machine.

**Table 14** presents an accuracy comparison of three feature extraction methods, i.e., a full set of deep features extracted from InceptionV3 and DenseNet201 CNNs, a reduced set of features selected by the proposed GWO algorithm, and a standard genetic algorithm. The table demonstrates that the GWO algorithm achieves a better or comparable accuracy as compared to the other two feature selection methods with a significantly small feature set.

**Table 14:** Performance comparison of leukemia classification using three feature selection approaches, i.e., proposed GWO feature selection algorithm, feature selection using genetic algorithm, and full feature set.

Classifier	Full feature set		Genetic algorithm		Grey Wolf Algorithm	
	No. of features	Accuracy %	No. of features	Accuracy %	No. of features	Accuracy %
KNN cosine	3,986	97.2	1,520	96.2	797	97.9
KNN coarse		96.9		94.3		97.6
KNN cubic		98.1		97.2		97.4
KNN fine		95.1		96.1		96.4
SVM Gaussian		98.5		97.58		98.14
SVM regression		90.2		89.2		85.2
NN wide		96.2		94.5		95.8
Decision tree medium		84		81.2		82.4

KNN, K-nearest neighbor; SVM, support vector machine; NN, neural network.

In **Table 15**, a comparison is presented of the performance of our proposed method with some existing studies on leukemia identification. For a fair comparison, we selected the published studies that have used identical or almost similar datasets. Our proposed pipeline for leukemia binary detection and subtype identification achieves better or comparable performance metrics as compared to several other relevant studies with smaller feature sizes. This shows the validity and applicability of the proposed approach.

**Table 15:** Comparison of classification accuracy of proposed leukemia identification pipeline with some existing relevant works.

Work	Proposed method	Dataset	Disease type	Performance result
	Classification: SVM, ANN			
(24)	Multiscale blob detection deep feature extraction: AlexNet classification: SVM	ALL-IDB	ALL	Accuracy = 94.1%
(25)	Preprocessing segmentation: three-phase filtering morphological feature extraction	Self-collected	ALL	Specificity = 93.5%
(49)	Active contours for nucleus detection Shape and texture feature extraction classification: NN, SVM	Self-collected	Leukemia	Accuracy = 98.8%
(50)	Preprocessing Feature extraction: hybrid CNN Classification: bagging ensemble	ALL-IDB MiMMSBI SN-AM	ALL AML Multiple myeloma	ALL classification accuracy = 97.04%
This work	Contrast stretching using DE Deep feature extraction: InceptionV3 and DenseNet201 feature selection: GWO algorithm	ALL-IDB2 (40)	Leukemia ALL subtypes	Accuracy = 97.9% Accuracy = 98.14%

KNN, K-nearest neighbor; ANN, artificial neural network; ALL, acute lymphoblastic leukemia; NN, neural network; AML, acute myeloid leukemia; GWO, Grey Wolf Optimization.

## 6 Conclusion

Leukemia, a kind of hematologic malignancy, is frequently diagnosed in both pediatric and geriatric populations. An automated, computer-aided system of leukemia diagnosis is essential to aid medical professionals in making informed decisions about the disease and making an effective prognosis and treatment plan. In this work, we have demonstrated the effectiveness of deep feature optimization taking as a relevant design case, the detection, and classification of leukemia disease from blood smear images. We have proposed a hybrid deep learning methodology utilizing transfer learning as feature extraction. The problem of feature selection has been modeled as a combinatorial optimization problem and solved using a customized Grey Wolf Optimization algorithm. Our proposed leukemia identification system can be used as a supporting evidence tool in conjunction with other more detailed analysis methods such as RNA sequencing and molecular testing. We believe that the proposed expert system can also be integrated with more complex and rather practical image analysis systems such as image flow cytometry.

## Data availability statement

The original contributions presented in the study are included in the article/**Supplementary Material**. Further inquiries can be directed to the corresponding authors.

## Author contributions

MA: Conceptualization, Investigation, Methodology, Software, Writing – original draft, Writing – review & editing. MNA: Methodology, Software, Writing – review & editing. TA: Investigation, Methodology, Validation, Writing – review & editing. AA: Resources, Supervision, Writing – review & editing. MM: Resources, Supervision, Writing – review & editing. AM: Funding acquisition, Resources, Validation, Writing – review & editing.

## Supplementary material

Supplementary Table 1 Description of main symbols used in Algorithm 1.
