# Supplementary material for: An efficient decision support system for leukemia identification utilizing nature-inspired deep feature optimization
Source: Front Oncol. 2024 Mar 5;14:1328200. doi: 10.3389/fonc.2024.1328200 (PMC10949894; doi:10.3389/fonc.2024.1328200)
Supplement: Supplementary Table 1 — Description of main symbols used in Algorithm 1. [file Table_1.pdf]

Symbol	Description	Symbol	Description
$\mathbb{F}$	Fused feature matrix	L	Label vector of training set
d_{max}	Total no. of fused features per image	t_{max}	maximum no. of Hybrid BWO Algorithm
n_p	Population size	$\vec{\mathbb{X}}$	Whale population matrix
$\vec{X}^*$	Iteration best individual	Γ^*	Iteration best fitness
$\vec{X}$	One binary individual of population matrix $\vec{\mathbb{X}}$	$\mathbb{F}_2$	Features extracted from $\mathbb{F}$
h_0	split ratio of training and texting parts of $\mathbb{F}$ for KNN	K	size of neighbors for KNN
a_c	classification accuracy	Γ	fitness value (error rate)
n_{pred}	No. of successfully predicted labels	n_{test}	total no. of test labels
p_r	Binomial Crossover probability Differential Evolution	$\vec{M}_i$	Mutation vector
$\vec{U}_i$	Trial vector	$\vec{C}_{i,j}$	Binomial Crossover vector
